# Brain blood flow pulse analysis may help to recognize individuals who suffer from hydrocephalus

**DOI:** 10.1007/s00701-023-05839-5

**Published:** 2023-10-27

**Authors:** Arkadiusz Ziółkowski, Magdalena Kasprowicz, Marek Czosnyka, Zofia Czosnyka

**Affiliations:** 1 Department of Biomedical Engineering, Faculty of Fundamental Problems of Technology, Wrocław University of Science and Technology, Wrocław, Poland; 2 Division of Neurosurgery, Department of Clinical Neurosciences, Addenbrooke’s Hospital, University of Cambridge, Cambridge, UK; 3 Institute of Electronic Systems, Faculty of Electronics and Information Technology, Warsaw University of Technology, Warsaw, Poland

**Keywords:** Transcranial Doppler, Cerebral blood flow velocity, Morphological analysis, Pulse shape analysis, Infusion test, Brain blood circulation

## Abstract

**Background:**

Normal pressure hydrocephalus (NPH) is often associated with altered cerebral blood flow. Recent research with the use of the ultrasonic method suggests specific changes in the shape of cardiac-related cerebral arterial blood volume (C_a_BV) pulses in NPH patients. Our study aims to provide a quantitative analysis of the shape of C_a_BV pulses, estimated based on transcranial Doppler ultrasonography (TCD) in NPH patients and healthy individuals.

**Methods:**

The C_a_BV pulses were estimated using TCD cerebral blood flow velocity signals recorded from probable NPH adults and age-matched healthy individuals at rest. The shape of the C_a_BV pulses was compared to a triangular shape with 27 similarity parameters calculated for every reliable C_a_BV pulse and compared between patients and volunteers. The diagnostic accuracy of the most prominent parameter for NPH classification was evaluated using the area under the receiver operating characteristic curve (AUC).

**Results:**

The similarity parameters were calculated for 31 probable NPH patients (age: 59 years (IQR: 47, 67 years), 14 females) and 23 healthy volunteers (age: 54 years (IQR: 43, 61 years), 18 females). Eighteen of 27 parameters were different between healthy individuals and NPH patients (*p* < 0.05). The most prominent differences were found for the ascending slope of the C_a_BV pulse with the AUC equal to 0.87 (95% confidence interval: 0.77, 0.97, *p* < 0.001).

**Conclusions:**

The findings suggest that in NPH, the ascending slope of the C_a_BV pulse had a slower rise, was more like a straight line, and generally was less convex than in volunteers. Prospective research is required to verify the clinical utility of these findings.

**Supplementary Information:**

The online version contains supplementary material available at 10.1007/s00701-023-05839-5.

## Introduction

Normal pressure hydrocephalus (NPH) is a neurological disorder primarily affecting older adults, characterized (among other features) by the accumulation of cerebrospinal fluid (CSF) in the brain’s ventricles and associated with progressive cognitive and motor dysfunction. Diagnosis of NPH typically involves a combination of clinical evaluation, brain imaging, and invasive tests such as the lumbar tap test and infusion testing to evaluate CSF dynamics [18, 31, 60]. According to the last guidelines for the management of idiopathic NPH in Japan [52], more than one symptom in Hakim’s triad [28] should be observed to suspect NPH. The incidence of the triad syndromes varies across studies: a gait disturbance exhibits 94–100% of NPH patients, cognitive impairment is present in 78–98% of NPH patients, and a urinary dysfunction affects 60–92% of NPH patients [24, 29, 41, 50, 67]. It was reported that a full triad was observed in approximately 60% of NPH-diagnosed patients [24, 35, 67], whereas a large-scale questionnaire study in Japan revealed that a complete triad was exhibited in only 12.1% of NPH patients [41]. The identification of the triad symptoms is an initial step in the further NPH diagnosis procedure. The next step is usually the assessment of ventriculomegaly based on CT/MRI images which are also not unified. Several parameters related to the ventricle’s size or shape are used in NPH diagnosis. The most frequently reported parameters are Evans’ index and callosal angle. A recently published meta-analysis revealed that the diagnostic performance expressed as the area under the ROC curve (AUC) was 0.87 (95% CI: 0.84–0.90) for Evans’ index and 0.97 (95% CI: 0.95–0.98) for callosal angle [55]. The threshold for both parameters is not unified and has been reported to be 0.3 [30, 71] or 0.32 [49] for Evans’ index and 90° [30, 46, 57, 61, 71], 100° [49], and 123° [10] for callosal angle. Another two parameters useful in MRI image evaluation are the brain-to-ventricle ratio and the convexity cistern to ventricle ratio. Their accuracy in differentiation between NPH patients and healthy individuals reported as AUC was equal to 0.97 and 0.96 for the brain-to-ventricle ratio and convexity cistern to ventricle ratio, respectively [72].

If Hakim’s triad and CT/MRI scan evaluation suggest the diagnosis of NPH, the CSF tap test or infusion study is often performed in order to assess the dynamics of CSF circulation and the probability of benefit from shunting [18, 31, 52, 60]. The tap test is an invasive procedure in which typically 40–50 ml of CSF is drained from the lumbar space [66]. According to a systematic review [48], the CSF tap test has a sensitivity of 58% (range 26–87%) and a specificity of 75% (range 33–100%). The positive response to the CSF tap test is an improvement in clinical symptoms after the test. However, the test is evaluated using different scores around the world [52]. Alternatively to (or together with) the CSF tap test, the infusion test is performed [18, 31, 52, 60]. The infusion test is more invasive than the tap test because it requires the injection of physiological saline or artificial CSF into the CSF space. During the injection, the intracranial pressure (ICP) is monitored, and the resistance to CSF outflow (R_CSF_) is calculated based on the pressure response to a controlled volume increase. The threshold for R_CSF_ is reported to be 13–18 mm Hg/ml/min with a positive predictive value between 80 and 92% [52]. It was also reported that analysis of slow waves of ICP, recorded during overnight monitoring [16, 59, 63, 64], and measurement of optic nerve sheath diameter [23] may be helpful additional measures in NPH diagnosis. However, the pathophysiology of hydrocephalus includes not only impaired CSF circulation and poor pressure–volume compensation but also the interference of abnormal CSF with cerebral blood flow (CBF) [19, 54]. A reduction in CBF associated with increased cerebrovascular resistance and decreased cerebrovascular compliance is frequently noted in NPH patients [4, 7, 8, 14, 27, 38, 39, 42, 45, 53, 65, 68]. The decrease in CBF observed in NPH is thought to result from increased CSF pressure and increased ventricular volume [26, 44, 51, 70], leading to cortical compression and stretching of blood vessels and white matter fibers [20, 22]. Another study also points out the role of parallel changes in cardiac function and systemic blood flow in the decrease of CBF in chronic hydrocephalus [21]. Moreover, underlying cerebrovascular disease is an important predictor of poor outcomes after the implantation of a hydrocephalus shunt [7]. Patients with cerebrovascular disease that prevails over disturbance in CSF circulation and poor pressure–volume compensation may not exhibit clinical improvement after shunting [15, 19].

Positron emission tomography (PET) and magnetic resonance imaging (MRI) can be used to assess alterations in cerebral blood circulation and cerebral blood volume; however, the downsides of these advanced imaging techniques are their high cost and low availability. In contrast, acoustic-based methods provide non-invasive, low-cost, real-time measures of cerebrovascular function. By transmitting short ultrasonic pulses from one side of the skull to another and dynamically measuring the time-of-flight of the pulses [56, 58], altered shapes of the cerebral arterial blood volume (C_a_BV) pulses have been observed in NPH-diagnosed patients. Following the successful treatment, the shape of the C_a_BV pulses became similar to those observed in healthy volunteers, suggesting it is a possible indicator of effective NPH treatment [12]. However, this method of measurement is still under development and is not yet available on the global market. We recently proposed an ultrasound-based method for assessing C_a_BV changes based on the cerebral blood flow velocity (CBFV) signal measured with a commonly available transcranial Doppler (TCD) device and modeling global cerebrovascular dynamics [37]. In the current study, we aim to analyze the shape of the pulse changes of C_a_BV in healthy volunteers and probable NPH patients using this methodology. We hypothesize that the shapes of C_a_BV pulses calculated from TCD measurements differ between healthy individuals and NPH patients and that a quantitative measure may help to non-invasively identify patients suffering from hydrocephalus.

## Methods

### Patient cohort

#### NPH

Thirty-one non-shunted elderly (age > 35 years) probable NPH patients were selected from a larger database of 42 patients who underwent constant rate infusion tests at Addenbrooke’s Hospital (Cambridge, UK) combined with TCD monitoring between 1992 and 2006. All patients had an Ommaya reservoir implanted to facilitate cerebrospinal fluid sampling without the need for repeated lumbar punctures during the diagnostic process. Additionally, if there was a clinical indication, the reservoir enabled overnight monitoring of ICP. The NPH was diagnosed by a neurosurgeon specializing in hydrocephalus management based on clinical symptoms (gait disturbance, cognitive impairment, and impaired bladder control) and CT/MRI imaging. All the subjects had clinical symptoms (at least two symptoms from Hakim’s triad [52]) and an increased Evan’s ratio (> 0.3 [30, 71]). Patient characteristics (age and sex) and values of CSF compensatory parameters calculated from the infusion test (mean ICP, R_CSF_, and brain elsticity (E)) are provided in the “Results” section. The authors of this study did not have access to additional clinical data such as raw CT/MRI images, the exact values of Evan’s index, and post-shunt outcomes. The primary selection criterium was a reliable, continuous recording of the CBFV signal at rest prior to the test (see the “Data processing” section for details about the signal inspection and signal reliability). Information on the patient’s age was missing in 8 cases; 3 patients were excluded due to the low quality of the CBFV signal, which was insufficient to analyze the CBFV pulse waveform in the time domain.

#### Healthy volunteers

From a database of 26 healthy volunteers for whom CBFV recordings were performed during spontaneous breathing at rest, 23 people were included in the final analysis. These data were collected at Wrocław University of Science and Technology (Wrocław, Poland) between 2014 and 2015. Inclusion criteria were age over 35 years, no smoking, absence of diseases of the nervous and cardiac systems, and medications known to affect cardiovascular parameters or CBF. The inclusion criteria were validated based on an interview before the recording. Three volunteers were excluded due to missing information on age.

### Data acquisition

#### NPH

In all patients, the infusion test was performed based on the methodology introduced by Katzman and Hussey [33]. The infusion study is a standard clinical investigation for NPH patients; thus, approval from the local ethical committee was waived. Additional non-invasive TCD monitoring during the test was approved by the local Ethics Committee in Cambridge (08/H0306/103). ICP was measured using a hypodermic needle (25 gauge) inserted in a pre-implanted Ommaya reservoir and connected to a pressure transducer via a saline-filled tube. The second needle was used for infusion. CBFV in the middle cerebral artery (segment M1) was monitored through the left or right transtemporal window using the TCD system (Neuroguard; Medasonics, Fremont, CA, USA) with a 2-MHz probe. The signals were recorded using custom software for waveform collection (WREC; W. Zabołotny, Warsaw University of Technology, Warsaw, Poland) and later by ICM+ (Cambridge Enterprise Ltd., UK). Each recording begins with a 5-min baseline preceding the infusion.

#### Healthy volunteers

The middle cerebral artery (M1) was insonated with TCD (Doppler BoxX, DWL, Compumedics Germany GmbH, Singen, Germany) through the left or right transtemporal window to capture CBFV. A 2 MHz ultrasound probe was attached to a plastic helmet and immobilized the volunteer’s head throughout the measurement. The CBFV signal was recorded with ICM+ software (Cambridge Enterprise Ltd., Cambridge, UK) for at least 5 min. The study was approved by the bioethical committee of Wrocław Medical University (permission no. KB-170/2014).

#### The recordings for both groups

NPH patients and healthy volunteers were curated by the person who did the measurement. The data curation consisted of reviewing the recording in the ICM+ software and marking artifacts (e.g., movement artifacts or signal interruptions), which reduced the final recording time for a part of the recordings to less than 5 min.

### Data processing

Prior to further analysis, all signals were visually inspected in the ICM+ to select good-quality recordings of the CBFV signal. The good-quality signal (sufficient to analyze in the time domain) has visible cardiac-related pulses and distinguishable at least two characteristic peaks: systolic and diastolic for at least half of the recording duration (distorted pulses, if any, were subsequently removed from the recording according to procedure described in supplementary materials). Moreover, the peaks and valleys of the pulses cannot be flattened (signal saturation), and the mean value cannot exceed the range of 30–120 cm/s (the range observed in healthy individuals and NPH patients [2, 3, 32, 40, 43]). In the group of NPH patients, the longest possible baseline periods prior to infusion were manually selected. In healthy volunteers, the whole reliable CBFV recordings were used for analysis. Values of E and R_CSF_ were calculated from infusion studies in probable NPH patients with the use of the ICM+ software [62].

Further, the following operations were performed:

Before pulse shape analysis, CBFV signals were up-sampled to the frequency of 200 Hz with simple cubic interpolation to increase their temporal resolution and enable precise detection of the beginnings of the pulses. The CBFV signals were then processed with an 8th-order Chebyshev type I digital low-pass filter with a cutoff frequency of 12 Hz to remove high-frequency noise. Individual pulse detection was performed using a modified Scholkmann algorithm [6]. C_a_BV pulses were then calculated from the CBFV signal based on the methodology described [1, 34, 36] and briefly presented in supplementary materials. The pulses were then detrended and normalized to values between 0 and 1 on the *X* and *Y* axes. To exclude the influence of heart rate on pulse-shape-related parameters, the *X*-axis was also normalized by linear interpolation to a uniform length of 200 samples. Finally, distorted pulses of C_a_BV were removed from the analysis (the exclusion criteria for pulse removal are described in supplementary materials), triangle similarity parameters were calculated for each legitimate C_a_BV pulse, and mean values of the parameters were calculated for each recording. Python 3.11 was used for all calculations.

### **Pulse C**_**a**_**BV triangle similarity parameters**

**Fig. 1:**
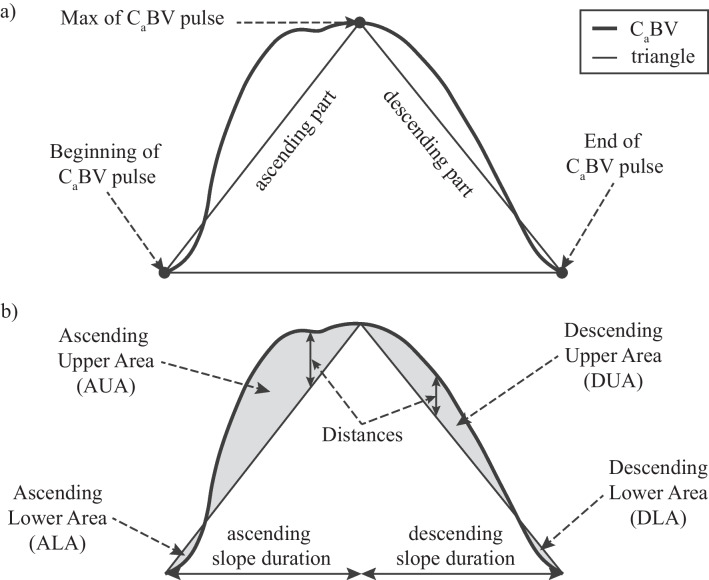
Visualization of **a** a triangle inscribed in a cerebral arterial blood volume (C_a_BV) pulse and **b** primary triangle similarity parameters

To quantify how the pulsation resembled a triangle, a virtual triangle was inscribed on the pulse. The triangle was defined by three points in the C_a_BV pulse: the beginning of the C_a_BV pulse (minimum value before the ascending slope of a pulse), the maximum value of the C_a_BV pulse, and the end of a pulse (minimum value after the descending slope of a pulse). By connecting these 3 points, a triangle was formed—see Fig. 1a.

Three basic types of parameters were proposed: (a) distances between the triangle and the C_a_BV pulse curve, (b) areas between the triangle and the pulse curve, and (c) durations of ascending and descending slopes. In total, 27 parameters were proposed. A detailed description of these parameters is presented in the Supplementary material. In general, the distance was calculated as the absolute difference between the values of the C_a_BV pulse and the triangular contour at a time point *t*. The area was calculated by summing all the distances contained in a given area. The duration of a slope was expressed as the time difference between the end of the slope and its beginning. The proposed parameters were calculated (a) separately for each area, (b) as the sums of the areas where the pulse contour is above or below the triangle, and (c) as the mean and max of the areas or distances. A visualization of selected triangle similarity parameters is shown in Fig. 1b. The list of all proposed parameters is presented in Table 1 in the Supplementary material.

### Statistical analysis

Mean values of ICP (for probable NPH patients) and CBFV (for all subjects) were calculated for each recording from the raw signals. Non-parametric tests were used for statistical analyses (the normality assumption was rejected by the Shapiro–Wilk test for the majority of variables). To eliminate the unequal influence of pulse-related parameter values in the statistics (due to the unequal number of pulses recorded for each patient or volunteer), the values of the parameters from each patient and volunteer were provided as a single mean value (calculated from all pulses belonging to a given patient or volunteer). The distributions of the mean values of physiological signals and other parameters, calculated as presented in the “Methods” section, were provided as the median and the upper and lower quartiles in the “Results” section. Differences in mean triangle-similarity parameters, age, and mean CBFV between probable NPH patients and healthy volunteers were tested with Wilcoxon’s signed rank test. The ROC curve and AUC were computed to evaluate the diagnostic accuracy of the most informative parameter for NPH classification. To select such a parameter, a machine learning predictive model—the decision tree classifier (described in [13]) of depth 1 with entropy as the optimization goal—was used. Spearman’s correlation coefficients were calculated to examine the relationship between the calculated parameters and age, mean ICP, and CSF compensatory parameters. The significance level was set at 0.05 for all analyses.

## Results

### Group characterization

The group of probable NPH patients included 14 females and 17 males with a median age of 59 years (IQR: 47–67 years). An age-matched group of healthy volunteers (age: 54 years (IQR: 43–61 years)) included 18 females and 5 males. There were no differences in age between these two groups (*p* = 0.100). CBFV was higher in healthy volunteers: 59.5 cm/s (IQR: 50.1–68.3 cm/s) than in probable NPH patients: 52.9 cm/s (42.4–62.0 cm/s) (*p* = 0.036). The ICP at the baseline prior infusion was 7.2 mm Hg (IQR: 4.4–9.5 mm Hg), R_CSF_ was 13.9 mm Hg/ml/min (IQR: 11.7–19.7 mm Hg/ml/min), and E was 0.18 1/ml (IQR: 0.12–0.30 1/ml).

### Lengths of the recordings in NPH and controls

The median length of CBFV recordings was 356 s (IQR: 291–410 s) in healthy volunteers and 331 s (IQR: 232–455 s) in probable NPH patients. The total number of pulses detected in recordings from 23 healthy volunteers and 31 NPH patients was 9666 and 14,923, respectively. Triangle similarity parameters were calculated for 8824 pulses recorded from healthy volunteers and 13,173 pulses from probable NPH patients.

### Differences in C_a_BV shape between healthy volunteers and probable NPH patients

**Fig. 2:**
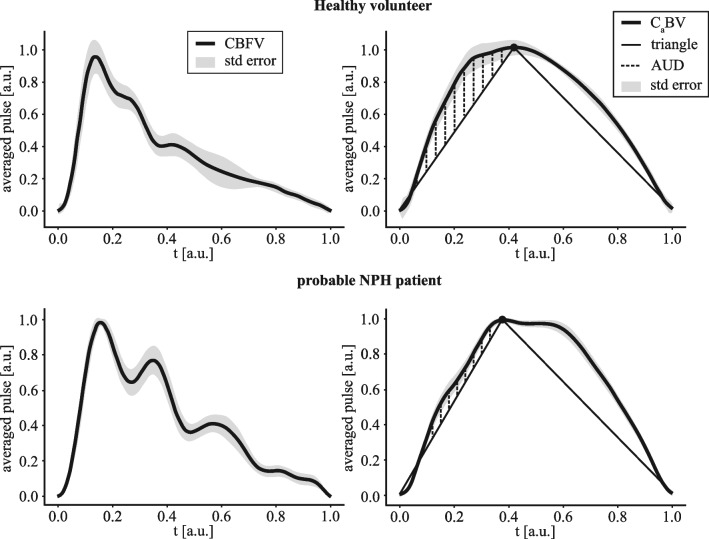
An example of averaged CBFV (cerebral blood flow velocity) and C_a_BV (cerebral arterial blood volume) pulses from recordings performed in a healthy volunteer (upper plots) and a probable NPH patient (normal pressure hydrocephalus, lower plots). Dotted lines visualize the ascending upper distances (AUD) as the most prominent differences in C_a_BV pulse shape between the healthy volunteers and NPH patients were observed for mean AUD

The analysis of the proposed descriptive parameters (see the “Methods” and “Data processing” sections) for each individual C_a_BV pulse waveform, revealed clear differences in values of 18 out of the 27 descriptors. The values of all these parameters are presented in Table 2 in the Supplementary material. Feature selection based on the decision tree classifier demonstrated that the most important parameter for discrimination in our dataset is mean ascending upper distance (mAUD), see Fig. 1—equivalent to mean ascending upper area. The AUC of the mAUD was 0.87 (lower and upper 95% confidence intervals: 0.77, 0.97, *p* < 0.001). This indicates thatthe ascending slope of the C_a_BV pulse was less convex, had a slower rise, and was more like a straight line in NPH than in volunteers. These differences are visualized in Fig. 2.

### Relationships between triangle similarity parameters and CSF compensatory parameters

C_a_BV shape-related parameters did not correlate with either R_CSF_, E, and mean ICP in NPH patients, or age in any of the group.

## Discussion

The results of this study support the hypothesis that the shape of the TCD-based pulse of C_a_BV differs between patients with NPH and healthy individuals. Our analysis revealed that the rising slope of the C_a_BV pulse in NPH patients was less convex and more like a straight line, resembling a triangle arm, while the pulse in healthy individuals had a more pronounced convexity.

The results are consistent with previous studies that have reported alterations in cerebral hemodynamics in NPH patients, including decreased cerebral blood flow, increased cerebral vascular resistance [8, 14, 27, 38, 39, 42, 45, 53, 65, 68], and decreased vascular compliance [4]. The mechanism underlying these changes is not fully understood, but it has been suggested that impaired drainage of CSF from the brain may result in the compression of small cerebral vessels [5, 25], leading to decreased CBF, which may also influence the shape of the C_a_BV pulse. Therefore, the possible mechanism for the observed changes in C_a_BV pulse shape can be explained that in healthy individuals, the cerebral vessels are able to rapidly accommodate changes in blood flow demand, resulting in a more pronounced convexity of the C_a_BV pulse. In contrast, in NPH patients, impaired cerebral venous drainage may lead to reduced vascular compliance and increased vascular resistance, which may limit the ability of cerebral vessels to rapidly accommodate changes in blood flow demand, resulting in a less convex ascending slope of the C_a_BV pulse.

Although the alterations in the shape of the C_a_BV pulse in NPH patients were previously reported by Chambers et al. [12], we cannot provide a direct comparison between their results and ours. Chambers et al. used a method based on the transmission of short ultrasonic pulses from one side of the skull to another and dynamic measurement of the time-of-flight of the pulses [56, 58]. This technique is not widely available. Whereas we used a global model of cerebral blood circulation and estimated C_a_BV pulses based on TCD measurement [36]. The shapes of the C_a_BV pulses differ between these two methods—the pulses assessed by Chambers et al. have three clearly distinguishable peaks (see Fig. 1 in [12]), whereas the C_a_BV pulses obtained with our method have barely visible peaks (see, for example, Fig. 2 or [11, 17, 34, 36, 69]). Therefore, Chambers et al. analyzed the heights of the detected peaks, and we proposed the quantitative similarity parameters. Nevertheless, our results are consistent with the results obtained by Chambers et al. [12] in the context of alterations in the C_a_BV pulse shape in NPH patients.

The proposed C_a_BV pulse analysis has several advantages. First, it uses a commonly available TCD device. The method is fully non-invasive and does not require the use of contrast agents or ionizing radiation, making it safe for repeated use. It has the potential to provide an objective and quantitative measure, which can improve the accuracy and reliability of diagnostic tests and may be especially useful in lower-income countries where the availability of MRI or computer tomography scanners is reduced.

Several limitations should be considered when interpreting the results of the present study. First, the groups of subjects were relatively small. In particular, signals from only 31 probable NPH patients and from 23 healthy individuals were analyzed. Thus, the findings should be interpreted with caution and confirmed in larger database. Second, the brain blood circulation model used in the study for the calculation of cerebral arterial blood volume was not directly compared with imaging modalities. Therefore, it is necessary to conduct further research to validate this model. Third, the custom-written algorithm for distorted pulse removal could exclude parts of reliable pulses from the analysis, but only a small percentage of pulses (10.5%) were rejected from the analysis as distorted. Fourth, the CBFV signals were up-sampled from 50 to 200 Hz to increase their temporal resolution, and a low-pass filtering with a cut-off frequency of 12 Hz was performed prior to analysis. It may have had a minor impact on the pulse shape—pulses became more smoothed (reduction of high-frequency noise), and both the pulse onset and the pulse maximum can be detected with width-augmented precision. It is possible that due to filtering, we lose important information from the high signal component, but both ICP and CBFV pulses are similar to some extent, and it was reported that the power of the ICP signal is mostly contained in the range below 8 Hz [9]. However, if this influence exists, it is systematic and fully reproducible. We have successfully applied the same up-sampling procedure and filter to CBFV recordings in our previous studies related to the shape of CBFV pulses [73, 74]. Fifth, we analyzed the diagnostic accuracy of NPH classification for only one, the most prominent C_a_BV shape-related parameter in our dataset. Studies conducted on larger cohorts are required to evaluate the diagnostic accuracy of this parameter and combinations of the proposed parameters. Sixth, we did not define the minimum length of the CBFV signal sufficient to evaluate the C_a_BV pulse shape-related parameters, which should be done in prospective studies. Finally, we did not find any significant correlation between the proposed parameters and CSF compensatory parameters. However, NPH is a heterogeneous disease often associated with changes in CBF, and CSF compensatory parameters themselves do not reflect the full picture of this complex disorder [47]*.*

## Conclusion

The findings suggest that the shape of the C_a_BV pulse waveform differs between healthy individuals and patients with NPH. Further research is needed to validate these findings and to determine the optimal parameters for C_a_BV pulse analysis in NPH diagnosis and treatment evaluation. However, the potential benefits of this methodology in terms of cost, accessibility, and safety makes it a promising avenue for clinical practice.

### Supplementary Information

Below is the link to the electronic supplementary material.

Supplementary file1 (PDF 467 KB)

## Data Availability

The data analyzed in this study are at present not publicly available. The dataset from the group of probable NPH patients is owned by Addenbrooke’s Hospital, Cambridge, UK. Requests to access these datasets should be directed to mc141@medschl.cam.ac.uk. The data from the group of healthy volunteers are owed by Wroclaw University of Science and Technology, Poland, and are available upon request to magdalena.kasprowicz@pwr.edu.pl.

## Abbreviations

*C*_*a*_*BV*: Cerebral arterial blood volume standardized by cross-sectional area of insonated artery [cm]
*CBF*: Cerebral blood flow [cm^3^/s]
*CBFV*: Cerebral blood flow velocity [cm/s]
*CSF*: Cerebrospinal fluid
*E*: Elasticity [1/ml]
*ICP*: Intracranial pressure [mm Hg]
*IQR*: Interquartile range
*MRI*: Magnetic resonance imaging
*NPH*: Normal pressure hydrocephalus
*PET*: Positron emission tomography
*R*_*CSF*_: Resistance to cerebrospinal fluid outflow [mm Hg/ml/min]
*TCD*: Transcranial Doppler
