# Supplementary material for: Brain blood flow pulse analysis may help to recognize individuals who suffer from hydrocephalus
Source: Acta Neurochir (Wien). 2023 Oct 27;165(12):4045–54. doi: 10.1007/s00701-023-05839-5 (PMC10739525; doi:10.1007/s00701-023-05839-5)
Supplement: Supplementary file 1 — Supplementary file1 (PDF 467 KB) [file 701_2023_5839_MOESM1_ESM.pdf]

SUPPLEMETARY MATERIALS

Brain blood flow pulse analysis may help to recognize individuals who suffer from hydrocephalus

Acta Neurochirurgica

Arkadiusz Ziółkowski[1*], Magdalena Kasprowicz[1], Marek Czosnyka[2,3], Zofia Czosnyka[2]

[1] Department of Biomedical Engineering, Faculty of Fundamental Problems of Technology, Wrocław University of Science and Technology, Wrocław, Poland

[2] Division of Neurosurgery, Department of Clinical Neurosciences, Addenbrooke's Hospital, University of Cambridge, Cambridge, United Kingdom

[3] Institute of Electronic Systems, Faculty of Electronics and Information Technology, Warsaw University of Technology, Warsaw, Poland

[*] Corresponding author

e-mail: arkadiusz.ziolkowski@pwr.edu.pl

Distorted pulses removing

Based on the visual inspection of all detected pulses, the following criteria were applied to the algorithm written in Python for the automatic removal of distorted pulses: 1) pulse shorter than 0.33 s or longer than 1.5 s (outside the physiological range of pulse length), 2) appearance time of the maximum CBFV pulse greater than 0.35 s (the maximum associated with a first or second peak of the CBFV pulse should appear within 0.3 s from the beginning of the pulse [1]), 3) the last

sample of a detrended and normalized C_aBV pulse greater than 0.15 (the minimum value of the detrended C_aBV pulse is expected at the end of the pulse, otherwise the pulse is detected incorrectly), 4) maximum triangle distance between triangle and C_aBV pulse curve greater than 0.65 (high amplitude of C_aBV oscillation are not expected in the C_aBV pulse, based on the shape of the pulse observed in MRI studies [2]).

C_aBV calculation

To calculate C_aBV pulses from the non-invasively measured CBFV signal the constant flow forward model of cerebral blood circulation was applied [3]. The main assumptions of this model are that: 1) cerebral blood outflow (CBF_{out}) is significantly less pulsatile than cerebral blood inflow (CBF_{in}) [4] and therefore CBF_{out} can be approximated by the mean value of CBF_{in} over a 6 seconds window [3] (see Supplement Figure 1a); 2) cerebral blood inflow can be estimated with CBFV multiplied by cross-sectional area of insonated artery (see equations in Supplement Figure 1a,b).

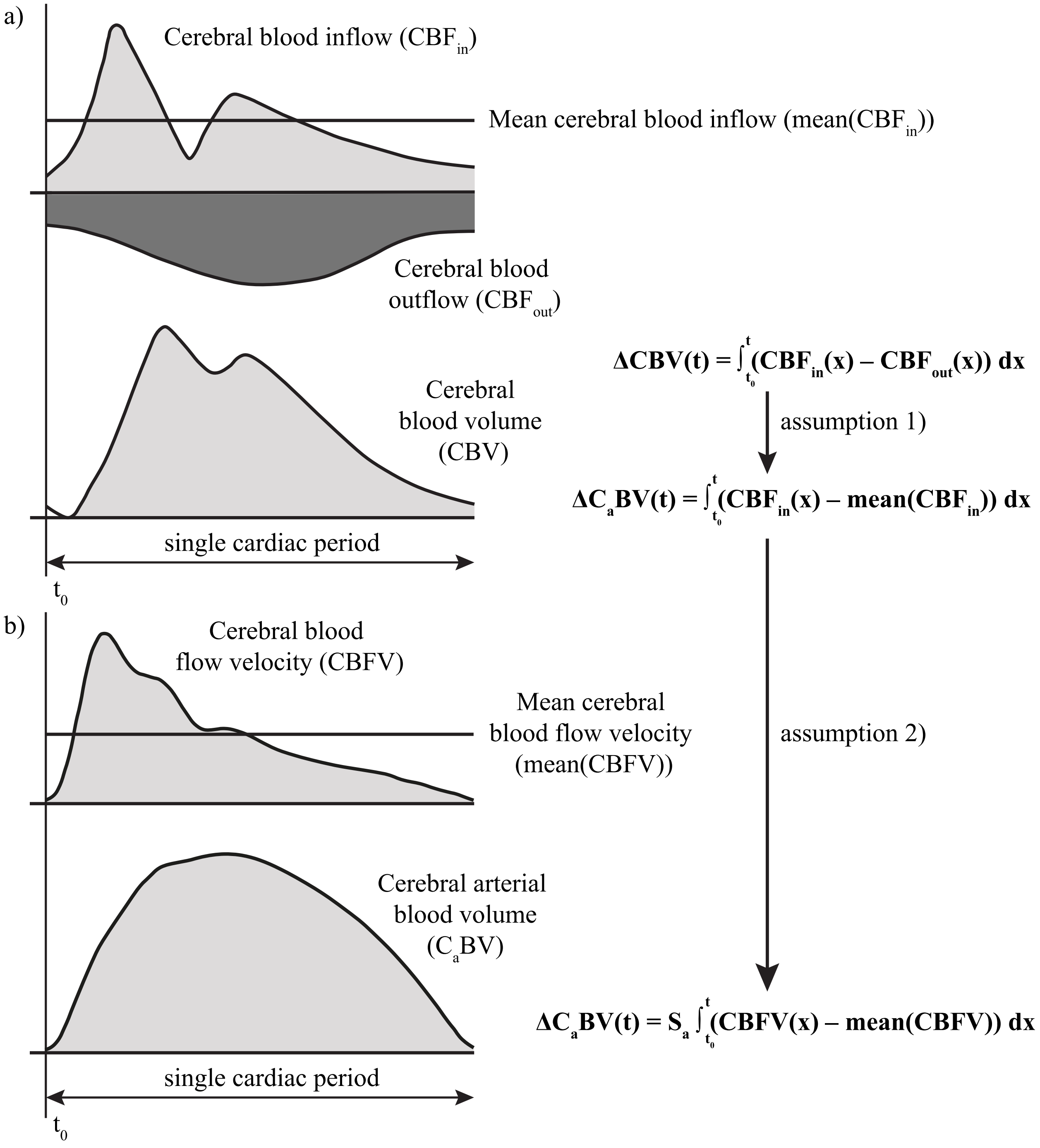

Supplement Figure 1. a): visualization of cerebral blood volume (CBV) calculated over a single cardiac cycle using invasively measured cerebral blood inflow and cerebral blood outflow (based on [4]). b): visualization of non-invasively estimated cerebral arterial blood volume (C_aBV) using constant flow forward model and TCD cerebral blood flow velocity signal. For the description of the assumptions see the content of Supplementary material. t_0 – beginning of a cardiac cycle, S_a – cross-sectional area of the insonated artery.

It was also assumed that the cross-sectional area of the insonated artery remains constant over a series of cardiac cycles [4], [5]. With these assumptions, the cross-sectional area of the insonated artery can be neglected in the equation in Figure 1b), and changes in C_aBV can be estimated using Equation (1).

$$\Delta C_a BV(t) = \int_{t_0}^{t} (CBFV(x) - mean(CBFV))\, dx\ [cm] \qquad (1)$$

where ΔC_aBV is the change in cerebral arterial blood volume during a single cardiac cycle, t_0 is the beginning of a single cardiac cycle, CBFV(x) is cerebral blood flow velocity at the moment t [cm/s], mean(CBFV) is a mean cerebral blood flow velocity calculated over a 6 seconds window [cm/s].

It is important to note that ΔC_aBV calculated in this way is normalized (divided) by the unknown cross-sectional area of the vessel S_a; therefore, units are not units of volume (cm^3) but cm and the value of ΔC_aBV cannot be compared between subjects. This however does not affect the shape of the C_aBV pulse, making the comparison analysis of the pulse shapes between healthy volunteers and NPH patients possible.

Supplement Table 1. List of all triangle similarity parameters that were analyzed.

Parameter name	Acronym	Parameter name	Acronym
**Total Area** – sum of all areas where the C_aBV pulse contour is above or below the triangle arm on both ascending and descending parts of the pulse	TA	**mean Ascending Lower Distance** – the average distance calculated from area where the C_aBV contour is below the triangle on ascending part of the pulse	mALD
**mean Distance** – the average distance calculated from all areas between C_aBV pulse contour and triangle arms	mD	**Maximum Ascending Lower Distance** – the maximum distance calculated from area where the C_aBV contour is below the triangle on ascending part of the pulse	MALD

**Maximum Distance** – the maximum distance from all areas between C_aBV pulse contour and triangle arms	MD	**Descending Upper Area** – the area where the C_aBV contour is above the triangle on descending part of the pulse	DUA
**Upper Area** – sum of areas where the C_aBV pulse contour is above the triangle arms	UA	**mean Descending Upper Distance** – the average distance calculated from area where the C_aBV contour is above the triangle on descending part of the pulse	mDUD
**mean Upper Distance –** the averaged distance calculated from all areas where the C_aBV pulse contour is above the triangle arms	mUD	**Maximum Descending Upper Distance** – the maximum distance calculated from area where the C_aBV contour is above the triangle on descending part of the pulse	MDUD
**Maximum Upper Distance –** the maximum distance from all areas where the C_aBV pulse contour is above the triangle arms	MUD	**Descending Lower Area** – the area where the C_aBV contour is below the triangle on descending part of the pulse	DLA
**Lower Area** – sum of areas where the C_aBV pulse contour is below the triangle arms	LA	**mean Descending Lower Distance** – the average distance calculated from area where the C_aBV contour is below the triangle on descending part of the pulse	mDLD
**mean Lower Distance –** the averaged distance calculated from all areas where the C_aBV pulse contour is below the triangle arms	mLD	**Maximum Descending Lower Distance** – the maximum distance calculated from area where the C_aBV contour is below the triangle on descending part of the pulse	MDLD
**Maximum Lower Distance –** the maximum distance from all areas where the C_aBV pulse contour is below the triangle arms	MLD	**Ascending Descending Duration Ratio** – the ratio of the duration of the ascending and descending part of the C_aBV pulse	ADDR
**Lower Upper Area Ratio** – the ratio of the sum of the areas where the C_aBV pulse is above the arms of the triangle to the sum of the areas where the	LUAR	**Frechet Distance** – distance between C_aBV pulse contour and the triangle arms calculated with the use of frechetdist 0.6 Python package, based on [6]	FD

C_aBV pulse contour is below the arms of the triangle.			
**Ascending Upper Area** – the area where the C_aBV contour is above the triangle on ascending part of the pulse	AUA	**Dynamic Time Wrapping Distance** – distance between C_aBV pulse contour and the triangle arms calculated with the use of dtw-python 1.3.0 package, based on [7]	DTWD
**mean Ascending Upper Distance** – the average distance calculated from area where the C_aBV contour is above the triangle on ascending part of the pulse	mAUD	**normalized Dynamic Time Wrapping Distance** – normalized distance between C_aBV pulse contour and the triangle arms calculated with the use of dtw-python 1.3.0 package, based on [7]	nDTWD
**Maximum Ascending Upper Distance** – the maximum distance calculated from area where the C_aBV contour is above the triangle on ascending part of the pulse	MAUD	**Maximum Appearance Time** – the time of appearance of the maximum value of the C_aBV pulse	MAT
**Ascending Lower Area** – the area where the C_aBV contour is below the triangle on ascending part of the pulse	ALA		

Supplement Table 2. Medians, lower (Q1) and upper (Q3) quartiles of all triangle similarity parameters. P-values are from the Wilcoxon signed rank test. Medians and p-values above 0.05 are bolded. Parameter names are given as acronyms, see Table 1 for full names.

	**Healthy volunteers n=23**			**NPH patients n=31**			
**Parameter**	**Median**	**Q1**	**Q3**	**Median**	**Q1**	**Q3**	**p-value**
TA	**26.45**	23.56	27.81	**22.47**	21.42	27.21	**0.025**
mD	**0.132**	0.118	0.139	**0.112**	0.107	0.136	**0.025**
MD	**0.290**	0.251	0.310	**0.253**	0.215	0.293	**0.017**
UA	**26.13**	23.19	27.57	**21.82**	20.54	27.08	**0.017**
mUD	**0.144**	0.129	0.150	**0.130**	0.122	0.145	**0.024**
MUD	**0.289**	0.251	0.308	**0.253**	0.215	0.293	**0.017**
LA	**0.374**	0.265	0.497	**0.661**	0.344	1.296	**0.005**
mLD	**0.017**	0.014	0.021	**0.026**	0.019	0.032	**0.002**
MLD	**0.033**	0.027	0.041	**0.050**	0.036	0.059	**0.003**
LUAR	**0.017**	0.008	0.030	**0.031**	0.016	0.061	**0.048**

AUA	**10.47**	9.775	14.16	**6.760**	5.497	8.870	<< **0.001**
mAUD	**0.153**	0.141	0.170	**0.114**	0.097	0.130	<< **0.001**
MAUD	**0.258**	0.237	0.296	**0.183**	0.152	0.239	<< **0.001**
ALA	**0.314**	0.200	0.452	**0.599**	0.322	1.241	**0.002**
mALD	**0.021**	0.017	0.025	**0.031**	0.023	0.038	**0.003**
MALD	**0.033**	0.026	0.040	**0.049**	0.035	0.059	**0.002**
DUA	**13.48**	12.91	15.08	**15.04**	12.96	17.21	0.157
mDUD	**0.128**	0.119	0.135	**0.131**	0.119	0.143	0.421
MUDD	**0.198**	0.187	0.213	**0.214**	0.186	0.230	0.151
DLA	**0.043**	0.036	0.060	**0.046**	0.020	0.103	0.958
mDLD	**0.005**	0.004	0.005	**0.004**	0.003	0.007	0.875
MDLD	**0.008**	0.006	0.009	**0.007**	0.004	0.011	0.875
ADDR	**0.802**	0.707	0.915	**0.688**	0.623	0.794	**0.002**
FD	**0.121**	0.110	0.140	**0.127**	0.104	0.155	0.916
DTWD	**1.277**	1.227	1.341	**1.244**	1.211	1.443	0.441
nDTWD	**0.003**	0.003	0.003	**0.003**	0.003	0.004	0.441
MAT	**431.7**	408.6	459.4	**398.9**	377.6	437.5	**0.010**

References:

[1] A. Ziółkowski *et al.*, “Peak appearance time in pulse waveforms of intracranial pressure and cerebral blood flow velocity,” *Front Physiol*, vol. 13, p. 2670, Jan. 2023, doi: 10.3389/FPHYS.2022.1077966/BIBTEX.

[2] A. Vallet *et al.*, “Biomechanical response of the CNS is associated with frailty in NPH-suspected patients,” *J Neurol*, vol. 267, no. 5, pp. 1389–1400, 2020, doi: 10.1007/s00415-019-09689-z.

[3] D. J. Kim *et al.*, “The monitoring of relative changes in compartmental compliances of brain,” *Physiol Meas*, vol. 30, no. 7, pp. 647–659, 2009, doi: 10.1088/0967-3334/30/7/009.

[4] C. J. J. Avezaat and J. H. M. van Eijndhoven, “The role of the pulsatile pressure variations in intracranial pressure monitoring,” *Neurosurg Rev*, vol. 9, no. 1–2, pp. 113–33, 1986, doi: 10.1007/BF01743061.

[5] M. Tóth, G. L. Nádasy, I. Nyáry, T. Kerényi, and E. Monos, “Are there systemic changes in the arterial biomechanics of intracranial aneurysm patients?,” *Pflugers Arch*, vol. 439, no. 5, pp. 573–578, Mar. 2000, doi: 10.1007/s004249900154.

[6] T. Eiter and H. Mannila, “ Computing discrete Fréchet distance. Tech. Report CD-TR 94/64,” Vienna, 1994.

[7] T. Giorgino, “Computing and Visualizing Dynamic Time Warping Alignments in R: The dtw Package,” *J Stat Softw*, vol. 31, no. 7, pp. 1–24, Aug. 2009, doi: 10.18637/JSS.V031.I07.
